# Laparoscopic gastrectomy using intracorporeally hand-sewn anastomosis of esophagojejunostomy, gastroduodenostomy, or gastrojejunostomy for gastric cancer

**DOI:** 10.1097/MD.0000000000019002

**Published:** 2020-01-31

**Authors:** Jia-Fei Yan, Ke Chen, Yu Pan, Hendi Maher, He-Pan Zhu, Song-Mei Lou, Yong Wang

**Affiliations:** a Department of General Surgery, Sir Run Run Shaw Hospital, School of Medicine, Zhejiang University,; b School of Medicine, Zhejiang University, Hangzhou, Zhejiang Province, China.

**Keywords:** hand-sewn, intracorporeal anastomosis, laparoscopic gastrectomy, stomach neoplasms

## Abstract

Laparoscopic gastrectomy (LG) using intracorporeal anastomosis has recently become more prevalent due to the advancements of laparoscopic surgical instruments. However, intracorporeally hand-sewn anastomosis (IHSA) is still uncommon because of technical difficulties. In this study, we evaluated various types of IHSA following LG with respect to the technical aspects and postoperative outcomes.

Seventy-six patients who underwent LG using IHSA for treatment of gastric cancer between September 2014 and June 2018 were enrolled in this study. We described the details of IHSA in step-by-step manner, evaluated the clinicopathological data and surgical outcomes, and summarized the clinical experiences.

Four types of IHSA have been described: one for total gastrectomy (Roux-en-Y) and 3 for distal gastrectomy (Roux-en-Y, Billroth I, and Billroth II). The mean operation time and anastomotic time was 288.7 minutes and 54.3 minutes, respectively. Postoperative complications were observed in 13 patients. All of the patients recovered well with conservative surgical management. There was no case of conversion to open surgery, anastomotic leakage, or mortality.

LG using IHSA was safe and feasible and had several advantages compared to mechanical anastomosis. The technique lengthened operating time, but this could be mitigated by increased surgical training and experience.

## 1 Introduction

Laparoscopic gastrectomy (LG) for gastric cancer has been employed for more than 2 decades. This procedure has undergone rapid development due to advantages including less pain, earlier recovery, better cosmesis, and comparable oncological outcomes.^[1–4]^ Surgical details such as the number and location of ports, lymph node dissection method, use or omission of mini-laparotomy, and surgical and oncological safety, were gradually established.^[5]^ However, the reconstruction methods, which are likely to affect the patients’ surgical outcomes and postoperative quality of life, have yet to be standardized. Intracorporeal anastomosis has advantages over an extracorporeal one because the former would create a smaller wound, provide a larger surgical workspace, and be less invasive.^[6,7]^ Generally speaking, the intracorporeal reconstruction methods can be classified into 2 main types: hand-sewn suturing anastomosis and stapled anastomosis using a linear or circular stapler.^[8]^ However, in our practice,^[8,9]^ it was found that there were certain limitations in mechanical approaches, compelling us to switch to manual reconstruction after sufficient laparoscopic experience was attained.^[10]^ This report contains the short-term outcomes of these methods of hand-sewn suturing intracorporeal anastomosis to assess its technical feasibility and discuss the advantages of this approach.

## 2 Methods

### 2.1 Patients

Between September 2014 and June 2018, 76 gastric cancer patients underwent LG using intracorporeally hand-sewn anastomosis (IHSA) in the Department of General Surgery, Sir Run Run Shaw Hospital. Patients were diagnosed with adenocarcinoma preoperatively and underwent LG, as well as modified D_2_ lymphadenectomy. We employ a multi-disciplinary team treatment model for every major abdominal surgery, during which the decision about surgical approaches are discussed. This is then presented to patients and their families to make a final decision. This research was approved by Zhejiang University's Ethics Committee, and written consent was obtained from all patients before surgery. American Joint Committee on Cancer (seventh edition) and TNM classification served as the criteria for clinical and pathologic staging. The severity of postoperative complications was based on the Clavien-Dindo classification.^[11]^

### 2.2 Surgical procedure

Details of lymphadenectomy are described elsewhere in our publications.^[9,12]^ Here, we describe the methods of hand-sewn suturing intracorporeal anastomosis after total (TG) or distal gastrectomy (DG).

**Figure 1:**
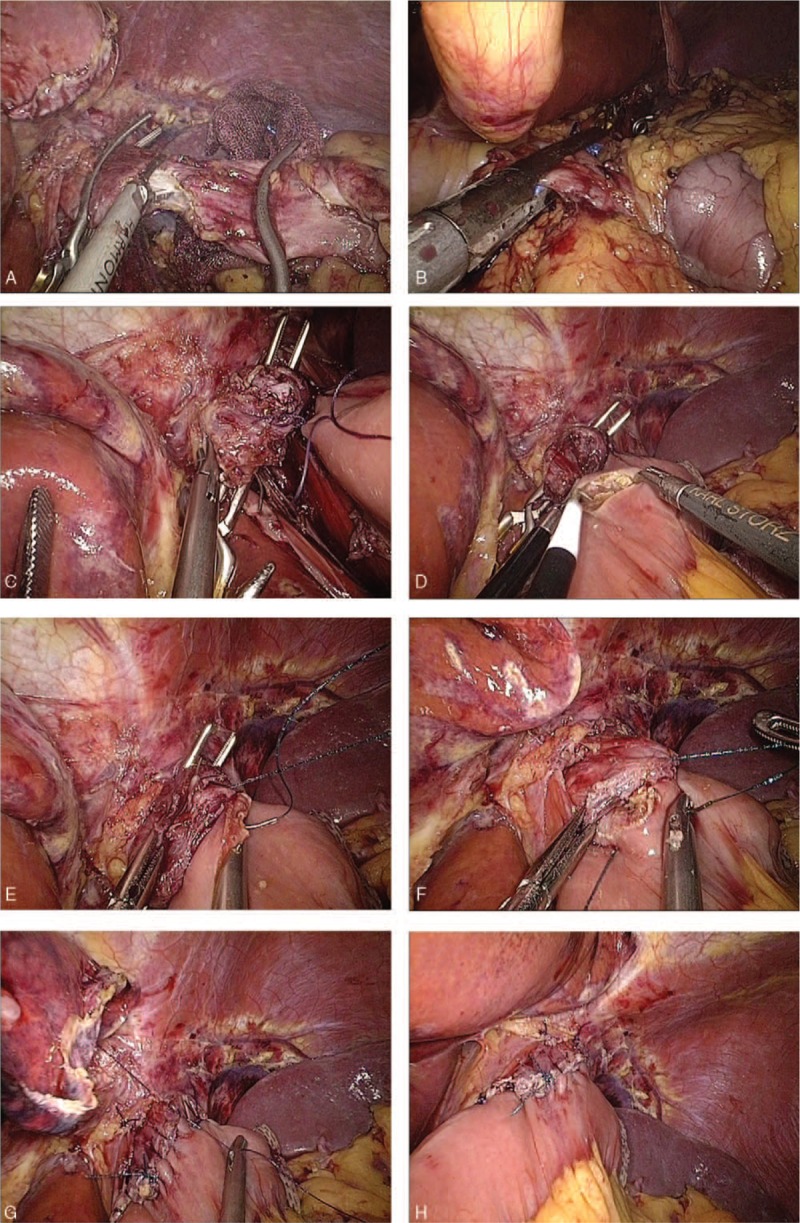
Intracorporeal hand-sewn end-to-side esophagojejunostomy. (A) Transection of the esophagus with ultrasonic coagulating shears between 2 clamps. (B) Transection of the duodenum with an endoscopic linear stapler. (C) The jejunum and esophageal stump attached to each other with seromuscular sutures. (D) A 2 cm wide incision at the antimesenteric side of the jejunum. (E) Suture of the posterior wall using continuous sutures. (F) Suture of the anterior wall using a continuous suture. (G) Strengthening of the seromuscular layer with interrupted sutures. (H) Complete esophagojejunostomy.

**Figure 2:**
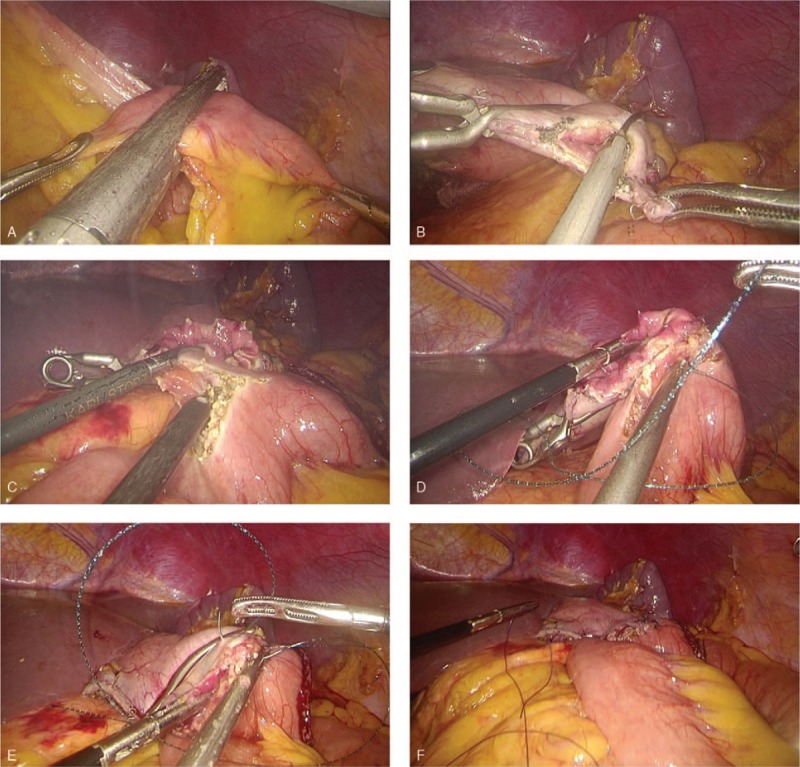
Intracorporeal hand-sewn end-to-side gastrojejunostomy. (A) Transection of the jejunum with an endoscopic linear stapler. (B) Transection of the gastric stump with ultrasonic coagulating shears. (C) A 3 to 4 cm wide incision at the antimesenteric side of the jejunum. (D) Suture of the posterior wall using interrupted sutures. (E) Suture of the anterior wall using a continuous suture. (F) Complete gastrojejunostomy.

**Figure 3:**
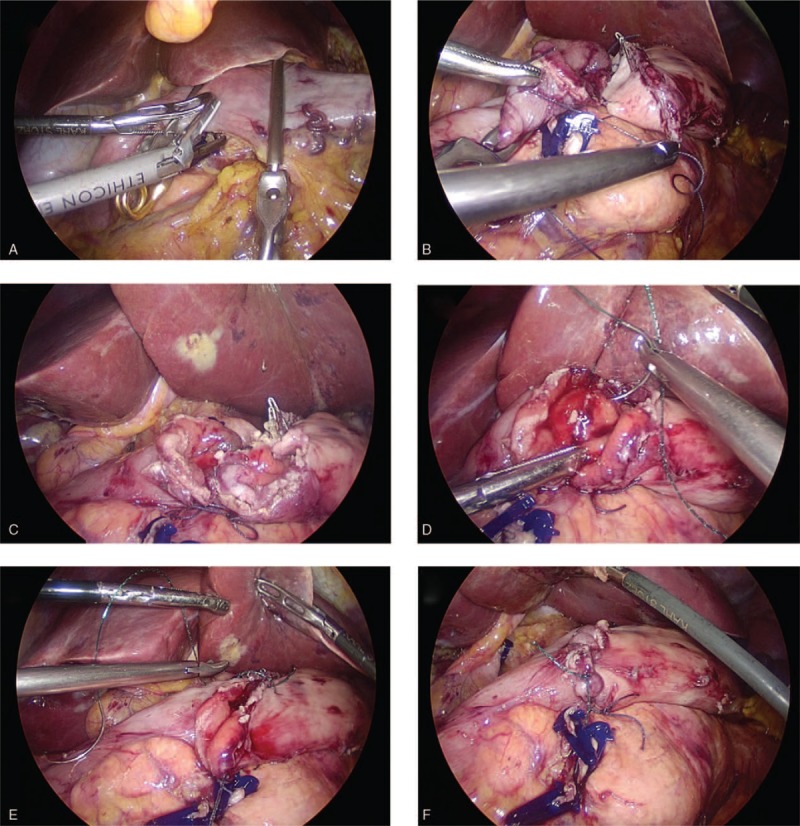
Intracorporeal hand-sewn end-to-end gastroduodenostomy. (A) Transection of the duodenum with ultrasonic coagulating shears between 2 clamps. (B) The duodenum and gastric stump attached to each other with seromuscular sutures. (C) Ready for anastomosis after transection of the gastric stump. (D) Suture of the posterior wall using interrupted sutures. (E) Suture of the anterior wall using a continuous suture. (F) Complete gastroduodenostomy.

**Figure 4:**
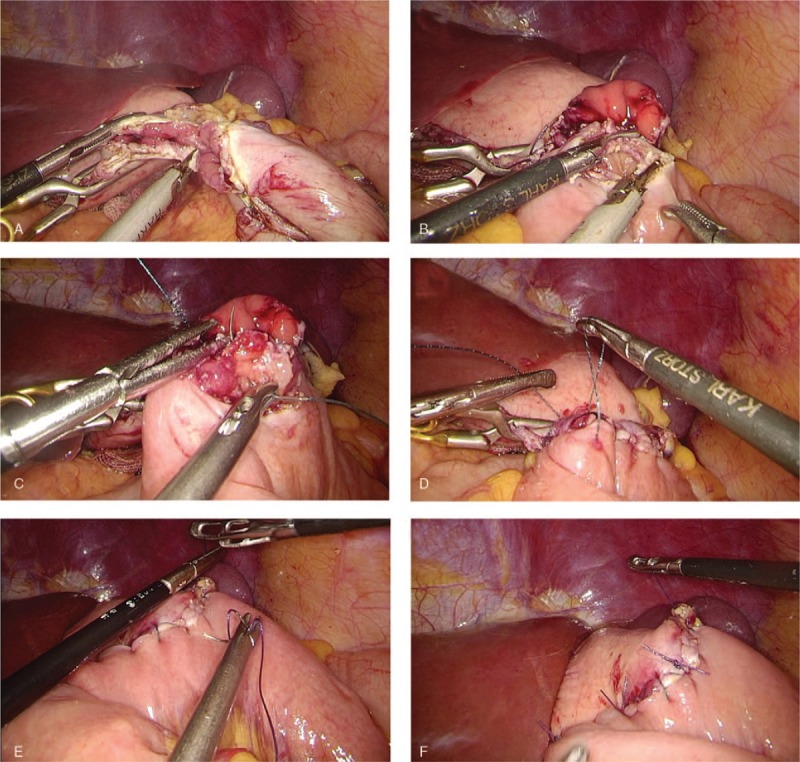
Intracorporeal hand-sewn end-to-side gastrojejunostomy. (A) Transection of the gastric stump with ultrasonic coagulating shears. (B) A 3 to 4 cm wide incision at the antimesenteric side of the jejunum. (C) Suture of the posterior wall using interrupted sutures. (D) Suture of the anterior wall using a continuous suture. (E) Strengthening of the seromuscular layer with interrupted sutures. (F) Complete gastrojejunostomy.

(1) Type A: esophagojejunostomy after TG (Roux-en-Y approach): 2 Endo Bulldog Clamps were placed at the cardia and distal esophagus to prevent contamination upon the completion of lymphadenectomy. Subsequently, the scissors or ultrasound scalpel were used to parallelly transect the esophagus between the 2 clamps (Fig. 1A). The jejunum was stapled at 20 cm to Treitz’ ligament, whereas the duodenum was transected by an endoscopic linear stapler at 3 cm from the pylorus (Fig. 1B). The jejunal loop was then created for introducing the esophageal stump to produce a reconstruction of end-to-side Roux-en Y after the frozen section confirmed a negative margin. The jejunum was also attached to the esophageal stump with several sutures interrupted with serosal muscularis at the esophageal stump's rear part (Fig. 1C). A matched enterotomy approximately 2 cm wide was created at the jejunum's antimesenteric side (Fig. 1D). The hand-sewn constant sutures were used to perform the esophagojejunostomy's posterior wall closure (Fig. 1E). Subsequently, the same constant sutures were used to suture the anterior wall (Fig. 1F). The interrupted sutures were utilized to strengthen the seromuscular layer (Fig. 1G). The enlarged umbilical incision was used to conduct a side-to-side jejunojejunostomy after esophagojejunostomy (Fig. 1H).
(2) Type B: gastrojejunostomy after DG (Roux-en-Y approach): The stomach was transected at a point 5 cm from the mass'superior margin with endoscopic linear staplers. The duodenum was divided at 1 cm distal to the pylorus after the dissection of lymph nodes. The jejunum was stapled at 20 cm from Treitz's ligament (Fig. 2A). An Endo Bulldog Clamp was put at the gastric stump's greater curvature side, which was later transected with ultrasonic coagulating shears (Fig. 2B). For approaching the gastric stump, the jejunal loop was introduced. Details of hand-sewn gastrojejunostomy were similar to Type A (Fig. 2C–F). Finally, through the umbilical incision which had been enlarged, a side-to-side jejunojejunostomy was performed.
(3) Type C: gastroduodenostomy after DG (Billroth I approach): The duodenum was parallelly transected with scissors or ultrasound scalpel between the 2 Endo Bulldog Clamps placed at the duodenum and pylorus (Fig. 3A). The stomach was divided as in the Type B procedure. The gastric stump was introduced to approach the duodenal stump. Subsequently, some sutures interrupted with serosal muscularis were placed at the duodenal and gastric stump's rear part (Fig. 3B). For end-to-end gastroduodenostomy, an incision 3 to 4 cm wide was made at the gastric stump's greater curvature side (Fig. 3C). The anterior wall was sutured with a continuous suture (Fig. 3D), while the posterior wall was sutured with interrupted sutures (Fig. 3E). Interrupted sutures were utilized to strengthen the seromuscular layer (Fig. 3F).
(4) Type D: gastrojejunostomy after DG (Billroth II approach): The resection approach and use of clamps is similar to the Type B procedure. The 15 cm distal portion of the jejunum loop to the ligament of Treitz‘s was introduced for approaching the gastric stump. Subsequently, several sutures interrupted with serosal muscularis were placed at the gastric stump and jejunum's rear part. An incision 3 to 4 cm wide was made at the jejunum's antimesenteric side for gastrojejunostomy (end-to-side). The details of hand-sewn gastrojejunostomy were very similar to the above-mentioned description (Fig. 4).

## 3 Results

### 3.1 Clinicopathological characteristics

The clinicopathological information is listed in Table 1. Of the 76 patients, 44 underwent TG and 32 underwent DG. There were 50 (66%) men and 26 (34%) women. The mean age was 58.3 years and the mean body mass index (BMI) was 22.4 kg/m^2^. Patients were classified as ASA 1 in 48 (63%) cases, ASA 2 in 24 (32%), and ASA 3 in 4 (5%). Mean tumor size was 4.1 cm. There were 35 stage I tumors (46%), 17 stage II tumors (22%), and 24 stage III tumors (32%).

**Table 1:**
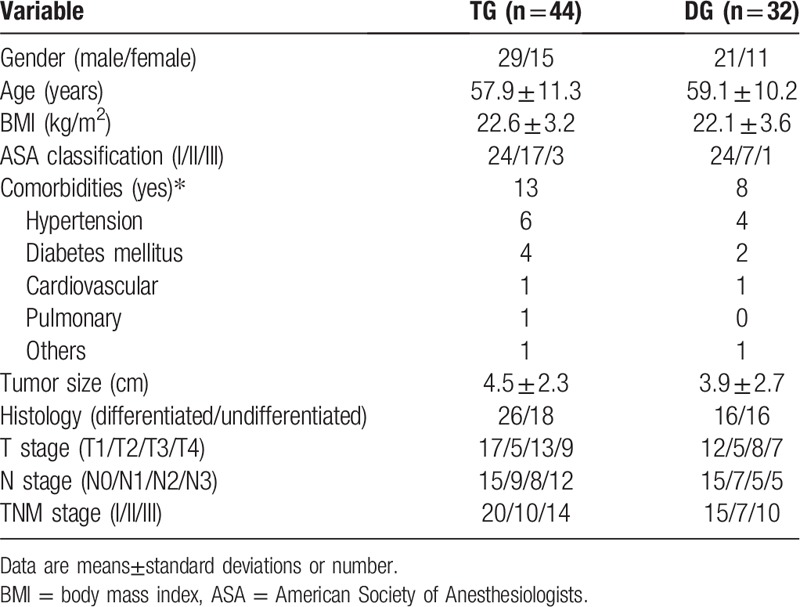
Clinicopathologic characteristics of patients.

### 3.2 Operative findings and postoperative clinical course

The operative findings and subsequent postoperative clinical course data are shown in Table 2. The types of anastomotic methods used were the following: Type A in 44 patients, Type B in 13 patients, Type C in 11 patients, and Type D in 8 patients. All procedures were successfully performed without intraoperative complications or conversion to open surgery. The mean operation time was 376.7 min (range: 230–420 minutes) and the mean intracorporeal anastomosis time was 81.5 min (range: 230–420 minutes). Mean blood loss was 82.7 mL (range: 50–200 mL). The mean number of retrieved lymph nodes was 34.3 (range: 24–69). The mean times to first flatus were 3.7 days (range: 2–7 days). The mean times to starting liquid and soft diets were 4.9 days (range: 3–7 days) and 6.6 days (range: 5–15 days), respectively. Finally, the mean postoperative hospital stay was 10.1 days (range: 7–20 days).

**Table 2:**
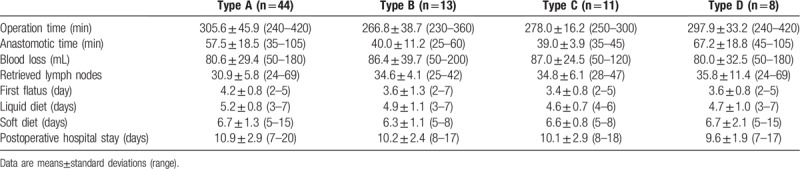
Operative findings and postoperative clinical course.

The overall morbidity was 17.1% (13/76 patients), and included intraluminal bleeding (n = 4), delayed gastric emptying (n = 1), abdominal abscess (n = 1), ileus (n = 3), pancreatic leakage (n = 2), wound infection (n = 1) and pulmonary infection (n = 1). No perioperative mortality was observed. Details of the postoperative complications and Clavien–Dindo classification are summarized in Table 3.

**Table 3:**
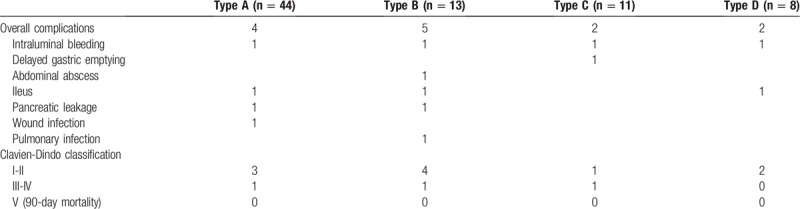
Postoperative complications.

## 4 Discussion

There were 2 basic approaches to the procedure. After finishing the lymphadenectomy laparoscopically, if a mini-laparotomy is made on epigastrium, through which the anastomosis is performed extracorporeally, this method is termed a “laparoscopic assisted gastrectomy” (LAG). The other approach, known as “totally laparoscopic gastrectomy” (TLG), is characterized by an intracorporeal anastomosis without auxiliary incision, thus preserving the integrity of the abdominal wall. There is evidence that TLG is superior to LAG in terms of better cosmesis, milder surgical trauma, and faster recovery.^[13–15]^ For intracorporeal anastomosis, various mechanically modified procedures have been devised, but an optimal method has not yet been established.^[5]^ There is a position that IHSA might be the most acceptable method, in contrast to the alternative mechanical procedures.^[16]^ However, IHSA requires greater skill. The acquisition of extensive skills in advanced laparoscopic surgery has allowed us to offer our patients the benefits of IHSA.

One of the most controversial areas of IHSA relates to the anastomosis-related complications. Anastomotic leakage is considered the most concerning cause of morbidity. The reported anastomotic leakage and stricture incidences of intracorporeally mechanical anastomosis after TG varied from 0 to 7.6% and 0 to 4.8%, respectively.^[17–20]^ The majority of studies investigating intracorporeally mechanical anastomosis after DG using mechanical anastomosis recorded anastomotic leakage rates less than 3%.^[21–24]^ The anastomotic leakage and stricture rates following mechanical anastomosis in our center were 2.0% and 6.0% in TG, respectively,^[8]^ whereas rates after DG were 0.5% and 0.0%, respectively.^[6]^ Encouragingly, no cases of anastomotic leakage or stricture were observed in this series when we had conducted IHSA following TG or DG (Table 3). Traditionally, nasogastric (NG) tubes were used for the prevention of anastomotic complications. In our experience, we have found that NG tubes often increase patient discomfort, without significant benefit. Therefore, we elected to place NG tubes only in a select group of patients. Specifically, those who suffered pyloric obstruction preoperatively were given an NG tube to relieve swelling.

Esophagojejunostomy was the most important step of reconstruction following TG. Several technical problems in the mechanical approach were found, such as exposure difficulties and weak points in double stapling. The utilization of a transorally inserted anvil had limitations including high cost, potential contamination, and injury to the esophageal mucosa.^[25]^ The side-to-side method, using a linear stapler, was restricted by the operative margins since a longer esophageal stump ought to remain.^[8]^ Regarding IHSA, it has several merits which are noteworthy:

(1) lower costs compared to any other mechanical approach,
(2) being performed more meticulously using magnified surgical vision,
(3) providing more tension-free anastomosis, thus avoiding injury to surrounding structures,
(4) requiring no excessive mobilization of the esophageal stump or Roux limb,
(5) allowing a relatively short esophageal stump, which benefits patients with insufficient margins,
(6) maintaining a favorable blood supply to the anastomotic stoma,
(7) negative margins can be confirmed before anastomosis.

The approaches of gastrointestinal anastomosis after DG are the same as standard laparotomy including Billroth I, Billroth II, and Roux-en-Y methods. These can be completed laparoscopically due to advancements of surgical instruments.^[5,8,9,26]^ Roux-en-Y was frequently referred to as the preferred procedure to prevent reflux gastritis and esophagitis. Disadvantages include surgical complexity and increased operative time. Additionally, if endoscopic linear staplers are used, the Roux-en-Y approach inevitably adds extensive costs. Mechanical Billroth I intracorporeally, which maintains physiological intestinal continuity, is commonly performed by Delta-shaped method.^[27]^ However, in addition to the advantage of technical simplicity, the procedure was limited by being unsuitable for obese patients or patients with large tumors in the low-to-mid stomach. Billroth II with a liberal anastomosis has been a more commonly used procedure than Billroth I in China. Since the majority of patients in this country present with advanced cancer, the length of the resection margins is considered an extremely important factor to ensure the adequacy of radical resection. We performed these 3 types of reconstructions by intracorporeal hand-sewn technique and documented satisfactory results. Several advantages, such as cost savings, maintenance of favorable blood supply, more liberal operation, which were acquired in IHSA esophagojejunostomy, also applied to IHSA anastomosis after DG. Nevertheless, we found the superiority of IHSA following DG was not as remarkable as was observed in the IHSA esophagojejunostomy, mainly because the gastric stump has better mobility in contrast to the esophagus. Considering that IHSA in DG is a more demanding and time-consuming technique, it was used less in our center.

Reconstruction time was another topic of concern. IHSA requires operators with significant experience in laparoscopic suturing skills. According to our experience, progressive practice (ie, practice on the simulator, then practice on animal models, followed by simple suture under laparoscopy, progressing finally to laparoscopic gastrointestinal anastomosis) can effectively shorten the learning curve. Meanwhile, the introduction of new instruments was helpful to master IHSA. Knotless barbed sutures were suggested as convenient and cost-effective instruments.^[28]^ We began using barbed sutures in 2014 and found them very helpful to steady tissues without the need for traction by a first assistant. This also helped with reducing surgical time, as the assistant could begin to explore the surgical cavity and facilitate the reconstruction process. The main published series of laparoscopic TG documented mean operative time of 240 to 380 minutes and reconstruction time around 50 minutes,^[25,29–31]^ whereas the series of laparoscopic DG recorded operative time of 200 to 280 minutes and reconstruction time around 30 minutes.^[9,27,32,33]^ Our series recorded a slightly prolonged (but acceptable) total time and anastomotic time of 305.6 and 57.5 minutes, respectively, in TG. The total time and anastomotic time in DG was 280.0 and 50.0 minutes, respectively. The Endo Bulldog Clamps were beneficial in preventing contamination from the contents of the gastrointestinal tract and facilitating the transection and removal of the specimen. In terms of the anastomosis course, work on the posterior wall was the most challenging step. Based on our experience, keeping the long corner stay sutures at the 3 o’clock and 9 o’clock positions of the anastomotic stoma allowed enough tension to provide a clear view of the posterior wall and allow more precise anastomosis. Lessening the anastomotic tension is also important to prevent leakage.

Limitations of this study included retrospective review, small sample size, single center design, and, most of all, lacking long-term outcomes due to a short follow-up period. Long-term quality of life (QOL) was considered as a critical outcome for all surgical treatments of gastric cancer, and IHSA may help to improve QOL.^[34,35]^ However, an accurate assessment of QOL should be conducted after a significant period of time after surgery. Therefore, the QOL evaluation of gastric cancer patients receiving IHSA would be our next task.

## 5 Conclusions

Intracorporeal anastomosis using hand-sewn sutures is a safe and feasible method for the treatment of gastric cancer. With an experienced laparoscopic suture technique, hand-sewn anastomosis is a promising and beneficial procedure.

## Author contributions

**Conceptualization:** Yu Pan.

**Data curation:** Yu Pan, He-pan Zhu.

**Funding acquisition:** Yong Wang.

**Investigation:** Ke Chen, Yu Pan.

**Methodology:** Song-mei Lou.

**Supervision:** Jia-fei Yan, Yong Wang.

**Writing – original draft:** Song-mei Lou.

**Writing – review & editing:** Jia-fei Yan, Hendi Maher.
